# Phenolic Profiles, Antioxidant Activity and Phenotypic Characterization of *Lonicera caerulea* L. Berries, Cultivated in Lithuania

**DOI:** 10.3390/antiox10010115

**Published:** 2021-01-15

**Authors:** Lina Raudonė, Mindaugas Liaudanskas, Gabrielė Vilkickytė, Darius Kviklys, Vaidotas Žvikas, Jonas Viškelis, Pranas Viškelis

**Affiliations:** 1 Laboratory of Biopharmaceutical Research, Institute of Pharmaceutical Technologies, Lithuanian University of Health Sciences, Sukileliu av. 13, LT-50162 Kaunas, Lithuania; mindaugas.liaudanskas@lsmuni.lt (M.L.); gabriele.vilkickyte@lsmu.lt (G.V.); vaidotas.zvikas@lsmuni.lt (V.Ž.); 2 Department of Pharmacognosy, Lithuanian University of Health Sciences, Sukileliu av. 13, LT-50162 Kaunas, Lithuania; 3 Lithuanian Research Centre for Agriculture and Forestry, Institute of Horticulture, Kauno str. 30, Babtai, LT-54333 Kaunas, Lithuania; darius.kviklys@lammc.lt (D.K.); jonas.viskelis@lammc.lt (J.V.); pranas.viskelis@lammc.lt (P.V.); 4 Department of Horticulture, Norwegian Institute of Bioeconomy Research—NIBIO Ullensvang, Ullensvangvegen 1005, NO-5781 Lofthus, Norway

**Keywords:** *Lonicera caerulea* L., honeysuckle berry, phenolic profile, anthocyanins, antioxidant activity

## Abstract

*Lonicera caerulea* L. is an early fruit-bearing plant that originates from harsh environments. Raw materials contain a body of different phenolic origin compounds that determine the multidirectional antioxidant and pharmacological activities. The aim of this study was to comprehensively evaluate the phenolic composition, antioxidant capacities, vegetative, pomological, and sensory properties and their interrelations of selected *L. caerulea* cultivars, namely ‘Amphora’, ‘Wojtek’, ‘Iga’, ’Leningradskij Velikan’, ‘Nimfa’, ‘Indigo Gem’, ‘Tundra’, ‘Tola’, and fruit powders. Combined chromatographic systems were applied for the qualitative and quantitative profiling of 23 constituents belonging to the classes of anthocyanins, flavonols, flavones, proanthocyanidins, and phenolic acids. The determined markers of phytochemical profiles were cyanidin-3-glucoside, rutin, chlorogenic, and 3,5-dicaffeoylquinic acid. Anthocyanins and the predominant compound, cyanidin-3-glucoside, were the determinants of antioxidant activity. Cultivars ‘Amphora’, ‘Indigo Gem’, and ‘Tundra’ contained the greatest total amounts of identified phenolic compounds. Phenotypic characterization revealed the superiority of cultivars ‘Wojtek’ and ’Tundra’ compared to other cultivars, although ’Wojtek’ had low phenolic content and antioxidant activity and ’Tundra’ got lower sensory evaluation scores. Coupling the results of phenotypic and phytochemical characterization, cultivar ‘Tundra’ could be suitable for commercial plantations.

## 1. Introduction

*Lonicera* L. genus (*Caprifoliaceae* Juss.) consists of about 200 species including ornamental, medicinal, and food plants [1]. One of the most economically important species is *Lonicera caerulea* L. (blue honeysuckle) bearing blue purple to dark blue fruits. The species is distributed in the Northern hemisphere, with main sites of origin in Russia, Canada, China, and Japan, growing in the continental climate zones Dwa, Dwb, Dwc, Dfb, Dfc climate regions [2,3,4,5]. The plant is highly resistant to various extreme environmental conditions and can successfully grow even in very harsh environments. An attractive trait is a fruit-bearing time, depending on the growing region, it occurs in May or June, the plant being one of the earliest available Northern fruits in the season [4,6,7]. The fruits, commonly known as berries (multiple fruits botanically) have been traditionally used as food and longevity sources in traditional local medicines. The fruits have been historically used to treat heart, eye, and gastrointestinal diseases [1,8]. Scientific research determined the health-promoting and therapeutic properties indicating cardioprotective, microcirculation improving, anti-diabetic, antibacterial, UV-photoprotective, chemopreventive, chemotherapeutic, anti-inflammatory, hepatoprotective, neuroprotective, and antioxidant effects of *L. caerulea* extracts [1,4,8]. Oxidative stress is strongly interrelated with inflammation, cancer, and other chronic degenerative diseases. The multidirectional antioxidant activity could interact with various oxidative stress implicated mechanisms, modulate the status of the cells, and express anti-inflammatory, degenerative preventive, or therapeutic effects [9]. Pharmacological effects are mainly determined by the body of distinct chemical origin bioactive compounds, namely, ascorbic acid, phenolic compounds, iridoids, triterpene compounds, carotenoids, fatty acids, and others [1,2,3,4,9,10].

*L. caerulea* fruits contain a significant amount of ascorbic acid with the amounts superior to other Northern fruits [2,11]. The phenolic fraction is constituted of hydroxycinnamic acids, anthocyanins, flavonols, flavones, flavan-3-ols. Their qualitative and quantitative profiles vary significantly, with the predominance of anthocyanins, flavonols, and caffeoylquinic acids [1,3,4,7,8,10]. Due to rich phytochemical composition blue honeysuckle fruits have been assigned to superfoods [3,9].

The qualitative anthocyanin profile is specific and characteristic to *L. caerulea* fruits, consisting of cyanidin-3-glucoside as the predominant compound (up to 90%) followed by cyanidin-3,5-diglucoside, peonidin-3-glucoside, and other minor anthocyanins [1]. The quantitative profiles vary significantly depending on horticultural cultivar, climatic and edaphic conditions of the growing area [2]. Auzanneau et al., 2018 determined that the growing year also affects the total amount of secondary metabolites [2]. Widespread species, growing in different climatic zones, possess differences in phytochemical composition within the same genotype [12]. Standardized growing conditions are in need to obtain chemically homogenous raw materials and are relevant for the sustainability of resources. The cultivated varieties occur from *L. caerulea* subspecies, the most common and easiest to grow being namely, ‘Tundra’, ‘Borealis’, ‘Aurora’, ‘Indigo’ series and ‘Boreal’ series. The research on cultivated blue honeysuckle is performed in Canada, Asia, and European countries and the cultivars differ in pomological characteristics, sensory properties, and phytochemical compositions [2,7,9,13,14,15,16]. The cultivars with determined phytochemical profiles and markers can be selected for standardized plantations [17]. Plantations have already been established in Russia and Japan, and the emerging research in other countries confirms the relevance. In Lithuania, the genetic research was performed on collection cultivars but no phytochemical profiling was performed up to date [18]. The evaluation of phenotypic traits, growing conditions, and coupling them to phytochemical profiles are essential for the production of the high-quality raw materials corresponding to the geo-authentic materials [12].

Blue honeysuckles contain small seeds that are imperceptible during consumption. Fruits can be easily freeze-dried, which ensures the retaining of color and bioactive compounds [6,19]. Freeze-dried powders are versatile with no waste technologies supporting products [20,21]. They can be easily incorporated in smart packaging indicating food spoilage due to pH changes and in functional food or other added-value products with notable antioxidant activity [4,22].

The aim of our study was to comprehensively evaluate the phytochemical composition, antioxidant capacities, vegetative, pomological, and sensory properties and their interrelations of selected *L. caerulea* cultivars (‘Amphora’, ‘Wojtek’, ‘Iga’, Leningradskij Velikan’, ‘Nimfa’, ‘Indigo Gem’, ‘Tundra’, ‘Tola’) fruit powders. The superior cultivars could be promoted for commercial crops in Dfb climatic regions with further functionalization of health-promoting products.

## 2. Materials and Methods

### 2.1. Plant Material

Vegetative growth parameters, yield, and fruit quality of eight *Lonicera caerulea* cultivars originated from Canada, Poland and Russia were investigated at the Institute of Horticulture, Lithuanian Research Centre for Agriculture and Forestry (Table 1) (55°4′55.67″, 23°47′53.99″ (World Geodetic System)). The plantation was established in 2016 on black woven mulch, planting distances were 3 × 1 m. A total of 10 plants of each cultivar were planted under a full randomized scheme.

Vegetative growth was evaluated by measuring shrub height (cm) and width (cm). Shrub density was evaluated on the 5-point scale, where 1—very sparse; 5—very dense. Shrub health status was evaluated on the 5-point scale, where 1—dying; 5—excellent status. Annual yield (kg) per individual shrub was recorded and accumulated yield (kg) during 2018–2020 is presented as an average of ten shrubs. Berries were harvested when their color was uniform and berries easily separated from the stalk. Average berry weight (g) was measured of the sample of 100 berries. The three-year average weight is presented. Berry shape was established according to descriptors. Fruit sensory evaluation was done by trained panelists in 2020. Average scores of 7 evaluations are presented.

### 2.2. Preparation of L. caerulea Extracts

Fruits were collected and immediately subjected to freeze-drying in a Zirbus lyophilizer (Zirbus Technology GmbH, Bad Grund, Germany) at 0.01 mbar pressure and −85 °C condenser temperature. The dried fruits were milled to powder and kept in a sealed container in a dark dry place. About 1 g (precise weight) of freeze-dried *L. caerulea* powder was weighted in a dark glass vial, with 10 mL of 80% (*v*/*v*) ethanol acidified with 2% hydrochloric acid. The extraction process continued for 40 min at 80 Hz and 904 W, in an ultrasonic bath (Elmasonic P, Singen, Germany). The extracts were filtered through 0.22 μm pore size membrane filters (Carl Roth GmbH, Karlsruhe, Germany) and transferred to the dark glass vials.

### 2.3. Chemicals

All the solvents, reagents, and standards used were of analytical grade and met all the set quality requirements. The following substances were used in the study: ethanol 96% (*v*/*v*) (AB Vilniaus degtinė, Vilnius, Lithuania), ABTS (2,2′-azino-bis(3-ethylbenzothiazoline-6-sulfonic acid), Trolox (6-hydroxy-2,5,7,8-tetramethyl-chroman-2-carboxylic acid), potassium persulfate, acetic acid, ammonia acetate, neocuproine (Scharlau, Sentmenat, Spain), TPTZ (2,4,6-Tris(2-pyridyl)-s-triazine) (Carl Roth, Karlsruhe, Germany), iron (III) chloride hexahydrate (Vaseline-Fabrik Rhenania, Bonn, Germany), acetonitrile, cyanidin-3-glucoside chloride, cyanidin-3,5-diglucoside chloride, cyanidin-3-rutinoside chloride, peonidin-3-glucoside chloride, cyanidin chloride, cyanidin-3-galactoside chloride, peonidin chloride, rutin, isoquercitrin, quercitrin, quercetin, isorhamnetin, apigenin, luteolin-7-O-glucoside, procyanidin B1, chlorogenic acid, caffeic acid, p-coumaric acid, ferulic acid, DMCA (4-(dimethylamino)-cinnamaldehyde), hydrochloric acid, (−)-epicatechin, sodium acetate trihydrate, copper (II) chloride dihydrate, formic acid (Sigma-Aldrich, Steinheim, Germany). During the study, we used purified de-ionized water prepared with the Milli–Q^®^ (Millipore, Bedford, MA, USA) water purification system.

### 2.4. Spectrophotometric Assays

#### 2.4.1. Determination of Total Proanthocyanidins

The procedure for the determination of total proanthocyanidins used in the present study is described in Heil et al., 2002 [23]. Briefly, 10 μL of *L. caerulea* fruit extract was mixed with 3 mL of 0.1% DMCA reagent dissolved in acidified ethanol (9 parts 96.3% ethanol and 1 part 36% hydrochloric acid). The reference solution was DMCA solution in acidified ethanol. After 5 min, the absorption of the test solution was measured with an M550 U*V*/*V* is spectrophotometer (Spectronic CamSpec, Garforth, UK) at a wavelength of 640 nm. The total amount of proanthocyanidins was calculated from a (−)-epicatechin calibration curve and expressed as mg/g (−)-epicatechin equivalent (EE) per one gram of dry weight (DW).

#### 2.4.2. Antioxidant Activity Assays

The ABTS assay was performed as described by Re et al., 1999 with some modifications [24]. Briefly, 3 mL of diluted ABTS radical cation solutions, produced by reacting 7 mM ABTS aqueous solution with 2.45 mM potassium persulfate and allowing the mixture to stand for 16 h in dark, were mixed with 20 μL of extracts. The decrease in absorbance was recorded at 734 nm after 1 h of incubation.

The ferric reducing activity (FRAP) was determined according to the method of Benzie and Strain (1996) [25]. During the evaluation, 3 mL of freshly prepared solutions of FRAP reagent, consisting of 300 mM acetate buffer, 10 mM TPTZ in 40 mM HCl, and 20 mM iron (III) chloride in a final ratio of 10:1:1 (*v*/*v*/*v*) were mixed with 20 μL of extracts, following by incubation for 1 h and absorbance recording at 593 nm.

The CuPRAC (cupric-reducing antioxidant capacity) assay was performed as described by Apak et al., 2007 [26]. In this assay, 3 mL of solutions of CuPRAC, consisting of 0.01 M copper (II) chloride, 0.001 M ammonium acetate buffer solution, and 0.0075 M neocuproine in a final ratio of 1:1:1 (*v*/*v*/*v*), were mixed with 20 mL of extracts, and the absorbance was measured at 450 nm.

All antioxidant activity measurements and calculations were performed using Trolox calibration curves and were expressed as µmol of the Trolox equivalent (TE) per one gram of dry weight, according to our previous research [27].

### 2.5. Chromatographic Assays

Quantification and profiling of phenolic acids, proanthocyanidins, flavonoids were performed using the validated high performance liquid chromatography (HPLC) method by Liaudanskas et al., 2014 [28] on a Waters 2695 chromatography system with a Waters 2998 PDA (photodiode array) detector (Waters, Milford, MA, USA). The chromatographic separation was performed on a YMC-Pack ODS-A C18 (250 × 4.6 mm; 5 μm) column equipped with a YMC-Triart C18 (10 × 3.0 mm; 5 μm) precolumn (YMC Europe GmbH, Dinslaken, Germany) at a constant temperature of 25 °C. The flow rate was 1 mL/min, injection volume—10 µL. Gradient elution (2% (*v*/*v*) acetic acid (A) and 100% (*v*/*v*) acetonitrile (B)): 0–30 min, 3–15% B; 30–45 min, 15–25% B; 45–50 min, 25–50% B; and 50–55 min, 50–95% B. The peaks were identified comparing retention times, spectral characteristics of analytes, and reference compounds.

The UPLC-ESI-MS/MS (ultra performance liquid chromatography with electrospray ionisation mass spectrometry) was performed using a previously described method by Gonzalez-Burgos et al., 2018 [29] using Waters ACQUITY UPLC^®^ H–Class (Waters, Milford, MA, USA) with a tandem quadrupole mass detector Xevo TQD (Waters, Milford, MA, USA), YMC Triart C18 (100 × 2.0 mm; 1.9 μm) column (“YMC”, Kyoto, Japan). Gradient elution (0.1%formic acid (A) and acetonitrile (B), flow rate 0.5 mL/min):A: initially 95%—1 min; to 70%—4 min; 50%—7 min; 95%—over 2 min. Collision energy and cone voltage were optimized for each compound separately. Retention times, compound molecular mass, and mass fragmentation were compared to literary data and reference compounds.

The variability in the qualitative and quantitative content of anthocyanins was evaluated by the validated method described by Vilkickyte et al. 2021 [30]. Chromatographic separation was performed with Waters ACQUITY Ultra-High-Performance LC system (Water, Milford, MA, USA) equipped with a photodiode array detector and an ACE Super C18 (100 × 2.1 mm, 1.7 μm) column (ACT, Aberdeen, UK). The gradient elution system consisted of 10% (*v*/*v*) formic acid in water (A) and 100% (*v*/*v*) acetonitrile (B), and separation was achieved using the following gradient: 0–2 min, 5–9% B; 2–7 min, 9–12% B; 7–9 min, 12–25% B; 9–10 min, 25–80% B; 10–10.5 min, 80% B; 10.5–11 min, 80–5% B; and 11.0–12.0 min, 5% B with flow rate 0.5 mL/min. The column was operated at a constant temperature of 30 °C and the injection volume was 1 μL. All anthocyanins were identified and quantified at 520 nm wavelength.

### 2.6. Statistical Analysis

Statistical analysis was performed using IBM SPSS 24.0 (SPSS Inc., Chicago, IL, USA) and Microsoft Office Excel 2017 (Microsoft, Redmond, WA, USA). All measurements were performed in triplicate, and results were expressed as mean ± standard deviation (SD). The one-way analysis of variance was performed, and post-hoc Tukey HSD multiple comparison test was used to identify significant differences at *p* ≤ 0.05. Radical scavenging and reducing activities were expressed as Trolox equivalent antioxidant activity as mean ± SD. Principal component analysis (PCA) was performed considering factors with eigenvalues higher than 1. Regression analysis was performed for the calibration curves of concentration-response. Correlations were evaluated using the Pearson correlation coefficient.

## 3. Results and Discussion

### 3.1. Evaluation of the L. caerulea Cultivars

The blue honeysuckle shrubs still did not reach their final size in the 5th year after planting (Table 2). Shrub height and width varied among cultivars from 87 up to 120 cm, and from 90 up to 110 cm, respectively. Though there were significant differences between cultivars both for shrub height and width, some more years are needed to draw final conclusions. The two cultivars ‘Amphora’ and ‘Leningradskij Velikan’ were distinguished by very dense shrubs; this is a negative character that bears difficulties during the berry harvest. Other tested cultivars did not differ significantly in shrub density. The two Polish cultivars, ‘Iga’ and ‘Tola’, were the same as the Canadian cultivar ‘Tundra’; in their 5th growing season, they had very healthy shrubs without any visual decline symptoms. ‘Leningradskij Velikan’ had the worst health status and the main negative symptoms were stunted new growth and leaf discoloration.

The highest cumulative yield was obtained of cultivar. ‘Tundra’ (Table 3). Cultivar ‘Wojtek’ also was very productive and did not differ significantly from cultivar ‘Tundra’. A total of five kg of berries harvested during three years, from 3–5 year old shrubs, were higher than the yield reported from the trials in Poland, where 3–4 years old cultivar ‘Wojtek’ gave around 1 kg yield [31], though other authors claim that 2 kg of berries from a shrub is an average yield for 5–6 year old shrubs [32,33]. In our trial, berries were harvested in one pick though there are recommendations to harvest in 3–4 times, or even up to 7 times, to prevent loss of overripe berries [34]. To consider that conditions cumulative yields in our trial could be increased by 10–15%. Cultivar ‘Amphora’ was the lowest yielding cultivar (1.8 kg during three years), and only cultivar ‘Nimfa’ did not differ significantly from it.

Average berry weight varied from 0.95 up to 1.19 g between cultivars (Table 2). Cultivars ‘Iga’ and ‘Wojtek’ had significantly larger fruits than most other cultivars tested. Such fruit size obtained in our trial is comparable to the results of some Polish and Slovenian trials [16,35].

Szot and Lipa (2013) reported a significant increase in average berry weight after the shrub pruning [36], but usually pruning starts from the 6th–8th year after planting of blue honeysuckle plantations, and our plants did not reach that age yet.

Sensory evaluation revealed significant differences between cultivars in berry appearance, flavor, and taste character (Table 4). The most attractive were berries of ‘Amphora’, ‘Wojtek’ and ‘Tola’. Berries of ‘Iga’ and ‘Tundra’ were evaluated to have a significantly lower total score. On the other hand, ‘Leningradskij Velikan’ berries had the best flavor score, possibly related to higher dominance of sweetness. The flavor score of the most attractive ‘Amphora’ and ‘Tola’ berries was the lowest, the same as ‘Iga’, which lead to lower overall evaluation. It is interesting that the taste character of all these cultivars was evaluated as sour or acid. Combining berry appearance and flavor, ‘Leningradskij Velikan’ had the highest ratings, whereas ‘Indigo Gem’, ‘Nimfa’ and ‘Wojtek’ did not differ significantly.

### 3.2. Phenolic Profiles of Fruits of Selected L. caerulea Cultivars

**Figure 1:**
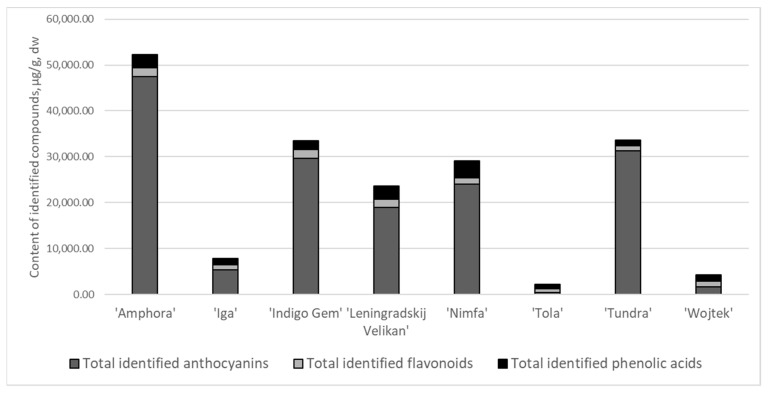
Content of total identified compound groups (µg/g DW) in *L. caerulea* fruits of different cultivars (flavonols, flavones and proanthocyanidns coupled to flavonoids).

Phytochemical profiles are determined by genetic origin, harvesting, and processing techniques of the plant materials and the environmental growing conditions. Environmental conditions are one of the main detrimental factors affecting the qualitative and quantitative compositions of plant materials. Central Europe is ascribed to the region of favorable growing conditions for *L. caerulea* species, as well as Canada and Northern countries of origin [2,9]. The HPLC-PDA and UPLC-PDA assays enabled profiling and quantification of anthocyanins, flavones, flavonols, and hydroxycinnamic acid contents in selected cultivars (Figure 1). Anthocyanins were the prevailing compounds and constituted from 38 up to 91% of total identified compounds, flavonoid fraction comprised from 3 up to 36%, and phenolic acids—3–42%.

The total amounts of identified anthocyanins varied significantly within the cultivars (Table 5). The greatest average amounts were determined in ‘Amphora’ (~48 mg/g), ‘Indigo Gem‘, ‘Nimfa’, ‘Tundra’, ‘Leningradskij Velikan’ ranging 19 to 31 mg/g, and the lowest amounts were determined for ‘Tola’, ‘Wojtek’ and ‘Iga’—0.46, 1.63 and 5.39 mg/g, respectively (*p* < 0.05).

The anthocyanin profile was predominated by the cyanidin-3-glucoside that accounted from 84 to 89% of total anthocyanins in cultivars ‘Wojtek’ and ‘Leningradskij Velikan’, respectively. The quantitative profiles of cyanidin-3,5-diglucoside, cyanidin-3-rutinoside, and peonidin-3-glucoside were cultivar specific, reaching up to 6, 8, and 4.5% of the total identified anthocyanins. The minor anthocyanins were up to 2%. The total content of the identified anthocyanins negatively correlated with the average fruit weight (R = −0.623; *p* < 0.05). The profile of anthocyanins generally occurs in the following manner: cyanidin-3-glucoside > cyanidin-3,5-diglucoside > cyanidin-3-rutinoside > peonidin-3-glucoside > pelargonidin-3-glucoside in L. *caerulea* genotypes [3,4,6,7,13]. Although certain authors indicate the presence of malvidin-3-glucoside, peonidin-3,5-diglucoside, pelargonidin-3-glucoside, cyanidin-3-gentiobioside, and acetylated derivatives [6,13,15]. The key marker of anthocyanin profile of L. *caerulea* fruits is cyanidin-3-glucoside accounting for 70–90% of total amounts [2,3,4,6,8,13,14]. The average contribution of cyanidin-3-glucoside in our tested cultivars were 86%, whereas the amounts of other identified anthocyanin derivatives were cultivar variant. The amounts of cyanidin-3-rutinoside tend to prevail after the cyanidin-3,5-diglucoside [6,37], but our results show, that only cultivars ‘Leningradskij Velikan’, ‘Wojtek’ and ‘Tola’ confirm this trend. Caprioli et al., 2016 determined cyanidin-3,5-diglucoside as the second prevailing compound in the spontaneously growing *L. caerulea* var. *kamtschatica* samples from Russia [3]. Khattab et al., 2017 determined the predominance of cyanidin-3-glucoside, peonidin-3-glucoside, and cyanidin-3,5-diglucoside in the profiles of Canadian cultivars ‘Tundra’ and ‘Indigo Gem’ [13]. Our results are in agreement confirming the second predominant compound peonidin-3-glucoside in ‘Tundra’ and cyanidin-3,5-diglucoside in ‘Indigo Gem’ samples. On the other hand, peonidin-3,5-dihexoside was the second prevailing compound, followed by peonidin-3-glucoside in ‘Tundra’ samples, cultivated in Slovenia [11]. The quantitative levels tend to be low temperature and solar radiation dependent [6,9]. Growing conditions affect the total amounts of anthocyanins in cultivars [2,3]. Cyanidin-3-glucoside as the prevailing compound in the phenolic fraction, determine most of the pharmacological effects of *L. caerulea* extracts [8,38,39].

Rutin was the phytochemical marker of the flavonoids ranging from 255.78 ± 8.38 µg/g in ‘Tola’ up to 779.31 ± 9.88 µg/g in ‘Indigo Gem’ cultivar. The other quantified flavonoid derivatives were isoquercitrin, quercitrin, quercetin, isorhamnetin, luteolin-7-glucoside, and apigenin, showing significant quantitative variation between tested cultivars. The flavonoid profiles of *L. caerulea* consist of (+)-catechin, (–)-epicatechin, quercetin, isorhamnetin, kaempferol, apigenin, and glycosides and aglycones of luteolin [6,7,11]. Rutin is the predominant compound in the flavonoid fraction and can be regarded as the phytochemical marker. These results are in agreement with various studies regarding the chemical variation of flavonoids [7,11,13,37]. In our study, Russian cultivars contained the greatest amounts of flavonoids in the following order ‘Nympha’ > ‘Amphora’ > ‘Leningradskij Velikan’. Gawronski et al., 2020 determined ‘Aurora’ with the highest amounts of flavonoids from the 30 tested *L. caerulea* cultivars [17]. Great variation in total contents of flavonoids was determined by various authors for the ‘Indigo Gem’, ‘Leningradskij Velikan’, ‘Nimfa’ cultivars due to different environmental conditions and growing techniques [17]. Anthocyanins and flavonoids are mainly located in skin tissues [6], therefore whole fruits or pomace materials could be selected for obtaining fruit powders for further functionalization [10].

The complex of hydroxycinnamic acids consisted of chlorogenic, caffeic, 4-*O*-caffeoylquinic, dicaffeoylquinic acids, *p*-coumaric, and ferulic acid. 3,5-dicaffeoylquinic acid prevailed in all tested cultivars (462.78–1975.90 µg/g), except ‘Tundra’ with the predominance of chlorogenic acid (676.87 ± 15.66 µg/g). Hydroxycinnamic acids are important precursors of flavor and they are highly abundant and characteristic in fruits of different genera [1,11,40]. Chlorogenic acid and 3,5-dicaffeoylquinic acid are principal hydroxycinnamic acids in *Lonicera* fruits [3,41]. The complex can also variably contain ferulic, caffeic, *p*-coumaric, neochlorogenic, and other caffeoylquinic derivatives [1,6,10]. Kucharska et al., 2017 and Ozmianski et al., 2016 determined three monocaffeoylquinic and three dicaffeoylquinic acids in *L. caerulea* fruits [7,10]. Liu et al., 2020 determined that chlorogenic acid together with cyanidin-3-glucoside and (+)-catechin inhibit α-amylase activity and gain perspective as a hypoglycemic functional ingredient [41]. Another group of compounds possessing antihyperglycemic effects are proanthocyanidins [42].

Significantly, the greatest total amount of proanthocyanidins, determined by spectrophotometric DMCA method, were determined in samples of cultivars ‘Amphora’ and ‘Leningradskij Velikan’ (2.16 ± 0.03 mg/g and 2.13 ± 0.01 mg/g, respectively) (Table 7). The amounts of procyanidin B1, determined by HPLC-PDA method, were well correlated with the total amounts of proanthocyanidins (R = 0.886; *p* < 0.05) and were highlighted in ‘Amphora’ and ‘Leningradskij Velikan‘, while ‘Tola’ and ‘Wojtek’ contained the lowest amounts (Table 4). A much higher total of proanthocyanidins levels, compared to detected procyanidin B1 amounts, indicate that there are much more unidentified proanthocyanidins in tested *L. caerulea* samples. Procyanidin dimers, trimers and up to polymers has been detected in the fruit samples by various authors [1]. Significant negative correlational interdependence was also determined between the total proanthocyanidins and average fruit weight (R = −0.602; *p* < 0.05). Proanthocyanidins in *L. caerulea* are also geographic origin-specific [6]. Their quantities are determined by the genotype and are negatively associated with the ripening [4]. The procyanidin dimers, trimers, and tetramers were determined in different cultivars [7,10,11]. Kucharska et al. study confirmed a great variation of amounts of individual and total proanthocyanidins in different cultivars [7]. The results are comparable with our tested cultivars.

The quantitative profiles were highly dependent on the cultivar. Overall, the cultivars ‘Amphora’, ‘Indigo Gem’, and ‘Tundra’ contained, significantly (*p* < 0.05), the greatest total amount of identified phenolic compounds (51.4, 32.6 and 33.0 mg/g, respectively), followed by ‘Leningradskij Velikan’ > ‘Iga’ > ‘Wojtek’ > ‘Tola’, the latter—only 1.7 mg/g.

The identity of flavonoids and phenolic acids in extracts of *L. caerulea* fruits was additionally confirmed by mass spectrometry, which data are presented in Table 6. Obtained mass fragmentation spectra, *m*/*z* proportions were identical with MS/MS data of reference compounds and literature.

### 3.3. Antioxidant Activity of L. of Fruits of Selected L. caerulea Cultivars

Antioxidant activity of phenolic rich plant materials is highly correlated with the antioxidant capacity, which depends on the structural peculiarities of compounds [27,43]. In this study, the antioxidant activity was evaluated using the in vitro techniques that differ in mechanism of action and experimental conditions. The radical scavenging activity was evaluated using ABTS in neutral medium, while reducing activity—FRAP (pH is acidic) and CuPRAC (pH—close to physiological values) assays. ABTS, FRAP, and CuPRAC belong to the single electron transfer based assays [44]. CuPRAC assay due to the electronic configuration of copper complex possess faster kinetics compared to FRAP assay [26].

The greatest radical scavenging activity was determined in the samples of cultivar ‘Indigo Gem’ (192.34 ± 27.95 µmol TE/g) followed by ‘Leningradskij Velikan’ > ‘Amphora’ > ‘Tundra’ > ‘Iga’ > ‘Tola’ > ‘Wojtek’ > ‘Nimfa’ (Table 7). Various studies determined cultivar ‘Nimfa’ with the lowest radical scavenging activities among 12 investigated cultivars [4,45]. The ferric reducing capacities were in a range of 107.66–707.60 µmol TE/g for the samples of cultivars in the following order—‘Tola’ < ‘Wojtek’ < ‘Leningradskij Velikan’ < ‘Iga’ < ‘Nimfa’ < ‘Amphora’ < ‘Tundra’ < ‘Indigo Gem‘. Cupric reducing capacities elucidated the highest (*p* < 0.05) antioxidant activity in cultivars ‘Amphora’ and ‘Nimfa’ (697.61 ± 30.86 µmol TE/g and 670.24 ± 42.02 µmol TE/g, respectively). Cultivar ‘Tola’ was distinguished with the lowest (*p* < 0.05) reducing activities. Higher antioxidant capacities have been determined for cultivars ‘Indigo Gem’ and ‘Tundra’, compared to other cultivars and certain fruits of *Vaccinium* and *Rubus* genus [4,13,27,46]. The three selected antioxidant evaluating methods, ABTS, FRAP, and CuPRAC, have revealed the multidirectional antioxidant capacity of L. caerulea fruit extracts and highlighted the superiority of the ‘Indigo Gem’ cultivar in terms of antioxidant activity. The applied antioxidant activity assays do not employ biological radicals, but, still, certain experimental setups can simulate conditions that can be met in food matrices of biological fluids, such as redox potential and pH [47]. Antioxidant activity mechanisms are highly interrelated with the anti-inflammatory, chemopreventive, cardioprotective, hepatoprotective, and neuroprotective activities [1,8,19]. The antioxidant activity of multi-phenolic compound containing plant matrices may differ due to their reaction kinetics, interactions in the sample, and peculiarities of activity enhancing structural elements [27,48].

Cupric reducing activities were well correlated with total proanthocyanidin content (R = 0.874; *p* < 0.05), and contents of procyanidin B1 (R = 0.738; *p* < 0.05), cyanidin-3,5-diglucoside (R = 0.810; *p* < 0.05), isoquercitrin (R = 0.952; *p* < 0.05), *p*-coumaric acid (R = 0.714; *p* < 0.05), chlorogenic (R = 0.881; *p* < 0.05), caffeic (R = 0.833; *p* < 0.05), 3,5-dicaffeoylquinic acid (R = 0.810; *p* < 0.05) and ferulic acid (R = 0.833; *p* < 0.05). In our previous research, we have determined caffeoylquinic acids as fast-acting antioxidant with high capacity [48]. Esters of hydroxycinnamic acids might be more active than free phenolic acids, they stabilize radical forms more efficiently [49]. The strong antioxidant properties of proanthocyanidins can be explained by the catechol moieties in their structure and the free phenolic hydroxyl groups. The coupling of monomers with 4β → 8 bonds provides structurally proper conditions for free radical inactivation and transition metal ion binding [50]. Zeng et al., 2020 determined the greatest antioxidant activity of procyanidin B2 consistently in various in vitro and in vivo models [51]. Ferric reducing activities correlated with total identified anthocyanins (R = 0.738; *p* < 0.05) and individual anthocyanins with the range of coefficients R = 0.762–0.905 (*p* < 0,05). The results are in agreement with the study of Moyer et al., 2002, that determined correlations between total anthocyanin content and FRAP results in the samples of *Vaccinium*, *Rubus* and *Ribes* fruits [52]. Radical scavenging activity was correlated with cyanidin-3-glucoside (R = 0.455; *p* < 0.05), cyanidin-3-rutinoside (R = 0.644; *p* < 0.05), rutin (R = 0.747; *p* < 0.05), and procyanidin B1 (R = 0.545; *p* < 0.05). Radical scavenging and reducing activities were well correlated with different groups of phenolics, indicating their versatile antioxidant potential. Correlation analysis suggested that anthocyanins and the predominant compound, cyanidin-3-glucoside, also proanthocyanidin B1, 3,5-dicaffeoylquinic acid, and rutin could be proposed as markers of reducing and radical scavenging activities of *L. caerulea* fruit extracts.

### 3.4. Principal Component Analysis of Fruits of Selected L. caerulea Cultivars

**Figure 2:**
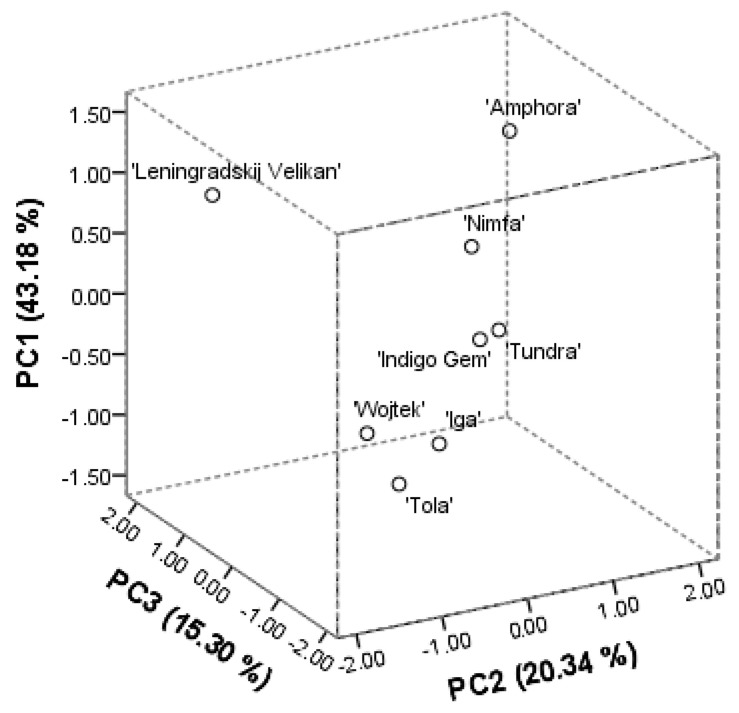
PCA score plots for the three principal components based on the phytochemical composition of *L. caerulea* fruits.

The principal component (PCA) analysis was performed on the phytochemical profile components, anthocyanins, flavones, flavan-3-ols, flavonols, proanthocyanidins, and hydroxycinnamic acids. The three main derived principal components explained 78.82% of the total variance. The score plot model has shown good separation between the investigated cultivars of *L. caerulea*. The PC1 was positively correlated with the amounts of identified anthocyanins, namely cyanidin (0.927), peonidin (0.956), cyanidin-3-glucoside (0.945), peonidin-3-glucoside (0.937), cyanidin-3-galactoside (0.915), cyanidin-3-rutinoside (0.836), cyanidin-3,5-diglucoside (0.799), and constituted 43.18%. The PC2 described 20.34% of the total variance and correlated positively with all the determined caffeoylquinic acids (0.675–0.918), procyanidin B1 (0.530). The PC3 accounted for 15.30% of the total variance and was well correlated with the amounts of procyanidin B1 (0.592) and flavonoid aglycones, namely apigenin, quercetin, (0.823, −0.958, respectively). The arrangements of score plots of investigated cultivars are shown in Figure 2. The first segregated group included ‘Wojtek’, ‘Iga’, and ‘Tola’ cultivars. They all were characterized by the lowest amounts of anthocyanins, proanthocyanidins, rutin, isoquercitrin, dicaffeoylquinic acids, and lowest or average reducing and radical scavenging activities. On the other hand, this group was distinguished with the greatest amounts of luteolin-7-*O*-glucoside, as well as, greatest fruit weight, and the highest score of appearance. The second group included ‘Indigo Gem’ and ‘Tundra’ cultivars. They possessed the greatest reducing activities in FRAP and above the average in CuPRAC assays, as well as high amounts of cyanidin-3-glucoside. Although, they had the lowest shrub height and fruit weight. Cultivars ’Leningradskij Velikan’, ‘Nimfa’, and ‘Amphora’ tended to be specific. Cultivar ‘Amphora’ distinguished with the highest amounts of anthocyanins and flavonol derivatives. Samples of the ‘Leningradskij Velikan’ cultivar were the richest in procyanidins, and flavonoid aglycones, namely apigenin, isorhamnetin. Both cultivars possessed high shrub width and the greatest density. Cultivar ‘Nimfa’ can be characterized by the greatest amounts of individual caffeoylquinic acids. The origin place of the cultivar determines the phytogeographical profile in a qualitative and quantitative manner. Numerous studies support information, that Russian cultivars contain higher amounts of phytochemicals [3,4,17]. In our study, the PCA analysis on phenolics clearly defined the cultivars into groups in the relation to their origin. The lowest (*p* < 0.05) total amounts of all groups of identified compounds, as well as, the lowest antioxidant capacities were determined for the Polish cultivars, namely ‘Wojtek’, ‘Iga’ and ‘Tola’. Genotype characterization resulted in the highest appearance and fruit weight scores. The Canadian cultivars ‘Indigo Gem’ and ‘Tundra’ were distinguished with the highest ferric reducing antioxidant power and low fruit weight. The Russian cultivars had the greatest anthocyanin contents and were specific in dominant phenolics of different chemical groups.

## 4. Conclusions

*Lonicera caerulea* fruit are attractive as polyphenolic containing materials with multidirectional antioxidant properties. The phenolic profiles are genotype variable and origin dependent. The key quantitative analytical markers of phytochemical profiles are cyanidin-3-glucoside, rutin, chlorogenic and 3,5-dicaffeoylquinic acid. These markers are suitable for the standardization of *L. caerulea* fruit preparations to control the quality and to ensure consistent, reproducible biological activity. The structural diversity of phenolic compounds in multicomponent plant matrices results in a very high potency antioxidant activity that was detected in all model systems. Cultivars ’Amphora’, ’Indigo Gem’, and ’Tundra’ contained the greatest total amounts of identified phenolic compounds. The Russian origin cultivars, namely, ’Amphora’, ’Leningradskij Velikan’ and ’Nimfa’ were specific in dominant phenolics, anthocyanins, proanthocyanidins, and caffeoylquinic acids, respectively. Tested cultivars showed significant differences in plant height, width, and density. All selected *L. caerulea* plants are suitable for growing in Lithuania, though cultivar characterization revealed the superiority of ’Wojtek’ and ’Tundra’ compared to other cultivars, although ’Wojtek’ had low phenolic content and antioxidant activity and ’Tundra’ got lower sensory evaluation scores. Coupling the results of cultivar and phytochemical characterization, cultivar ’Tundra’ could be suitable for commercial plantations. The obtained results could be used in further research evaluating chemotaxonomical significance, phytogeographical profiles, and in biological effect studies.

## Figures and Tables

**Table 1:** Cultivar origin and ripening time.

Cultivar	Origin	Time of Ripening
‘Amphora’	Canada	Early
‘Wojtek’	Poland	Medium early
‘Iga’	Poland	Medium early
‘Leningradskij Velikan’	Russia	Medium early
‘Nimfa’	Russia	Medium early
‘Indigo Gem’	Canada	Medium late
‘Tundra’	Canada	Medium late
‘Tola’	Poland	Late

**Table 2:** Shrub parameters and health status of blue honeysuckle cultivars, 2020.

Cultivar	Shrub Height, cm	Shrub Width, cm	Shrub Density *, (1–5 Scale)	Health Status
‘Wojtek’	120 a	100 b	4.3 b	4.6 b
‘Indigo Gem’	95 bc	90 b	4.1 b	4.0 cd
‘Iga’	120 a	100 b	4.0 b	5.0 a
‘Leningradskij Velikan’	100 bc	110 ab	5.0 a	3.7 d
‘Nimfa’	107 ab	95 b	4.0 b	4.5 bc
‘Amphora’	105 ab	110 ab	5.0 a	4.2 c
‘Tola’	100 bc	110 ab	4.1 b	4.9 ab
‘Tundra’	87 c	128 a	4.3 b	5.0 a

* 1—very sparse; 2—sparse; 3—medium dense; 4—dense; 5—very dense; Different letters represent statistically significant differences (*p* < 0.05) between values within the same column.

**Table 3:** Cumulative yield and fruit characteristics of honeysuckle cultivars, 2018–2020.

Cultivar	Cumulative Yield, kg/Shrub	Average Berry Weight, g	Berry Shape
‘Wojtek’	4.84 ab	1.15 a	oval
‘Indigo Gem’	3.08 cd	0.96 b	oval prolonged
‘Iga’	4.34 b	1.19 a	oval
‘Leningradskij Velikan’	3.22 c	1.05 ab	prolonged
‘Nimfa’	2.37 de	0.95 b	prolonged
‘Amphora’	1.78 e	0.95 b	prolonged
‘Tola’	4.23 b	1.02 ab	oval
‘Tundra’	5.21 a	0.98 b	oval rounded

Different letters represent statistically significant differences (*p* < 0.05) between values within the same column.

**Table 4:** Sensory characteristics of honeysuckle cultivars, 2018–2020.

Cultivar	Appearance	Flavor	Total Score	Taste Character
‘Wojtek’	4.64 ab	4.23 bc	4.38 abc	sour sweet
‘Indigo Gem’	4.53 b	4.59 ab	4.55 ab	tangy
‘Iga’	4.45 bc	4.02 c	4.15 de	sour sweet
‘Leningradskij Velikan’	4.01 d	4.93 a	4.61 a	sweet
‘Nimfa’	4.29 c	4.53 ab	4.45 abc	sweet tangy
‘Amphora’	4.69 a	3.89 c	4.32 bcde	acid
‘Tola’	4.62 ab	3.86 c	4.28 cde	acid tangy
‘Tundra’	4.02 d	4.21 bc	4.08 e	sweet tangy

Different letters represent statistically significant differences (*p* < 0.05) between values within the same column.

**Table 5:** Contents of individual phenolic compounds (µg/g, DW) in cultivars *of L. caerulea fruits*.

Compounds	‘Wojtek’	‘Indigo Gem’	‘Iga’	‘Leningradskij Velikan’	‘Nimfa’	‘Amphora’	‘Tola’	‘Tundra’
Cyanidin-3-glucoside	1372.44 ± 93.75 c	25833.20 ± 3476.11 b	4655.89 ± 247.85 c	16902.72 ± 263.25 b	20784.10 ± 2407.99 b	41162.46 ± 6694.48 a	394.55 ± 1.91 c	27587.45 ± 34.70 b
Cyanidin-3,5-diglucoside	44.31 ± 3.30 d	1220.38 ± 325.31 b	269.45 ± 14.74 d	389.00 ± 5.66 c,d	1432.43 ± 151.67b	2114.31 ± 325.20 a	19.00 ± 1.56 d	962.22 ± 8.80 b,c
Cyanidin-3-rutinoside	130.80 ± 6.10 c,d	885.93 ± 210.40 a,b	213.19 ± 8.00 c,d	852.25 ± 9.38 a,b	498.50 ± 53.45 b,c	1205.55 ± 228.71 a	24.73 ± 0.35 d	718.23 ± 2.51 b
Peonidin-3-glucoside	38.83 ± 0.80 d	1057.39 ± 256.94 b,c	180.63 ± 0.17 d	716.85 ± 11.12 c	1082.38 ± 30.01 b,c	1967.06 ± 249.65 a	18.73 ± 0.48 d	1326.58 ± 4.19 b
Cyanidin	30.73 ± 0.26 b	576.28 ± 250.24 a	41.14 ± 0.36 b	96.88 ± 1.34 b	137.32 ± 12.30 b	930.33 ± 169.83 a	3.77 ± 0.04 b	603.93 ± 2.18 a
Cyanidin-3-galactoside	6.86 ± 0.13 f	39.51 ± 0.11 c	10.41 ± 0.27 e	33.58 ± 1.36 d	40.89 ± 0.66 c	101.02 ± 0.84 a	ND	54.51 ± 0.48 b
Peonidin	48.37 ± 1.46 e,f	27.70 ± 2.29 c	17.70 ± 0.70 d	8.31 ± 0.69 e	22.72 ± 2.00 c,d	48.37 ± 1.46 a	1.93 ± 0.02 f	42.40 ± 0.98 b
Rutin	503.56 ± 24.22 c,d	779.31 ± 9.88 a	352.60 ± 13.25 e	549.27 ± 14.48 b,c	449.77 ± 9.36 d	586.43 ± 6.99 b	255.78 ± 8.38 f	355.96 ± 14.52 e
Isoquercitrin	36.82 ± 0.90 e	55.40 ± 0.99 d	90.94 ± 3.86 b,c	139.11 ± 5.40 a	100.69 ± 3.72 b	138.43 ± 5.74 a	26.30 ± 0.49 e	80.80 ± 1.18 c
Quercitrin	14.95 ± 0.50 c	13.36 ± 0.46 c	9.41 ± 0.12 d	26.04 ± 0.87 a	7.03 ± 0.17 d	21.30 ± 0.74 b	21.95 ± 0.72 b	12.24 ± 0.38 c
Quercetin	134.13 ± 11.64 a	143.30 ± 13.78 a	130.59 ± 13.17 a	124.21 ± 14.38 a	115.25 ± 9.65 a	47.25 ± 3.22 a	114.52 ± 7.35 a	128.78 ± 11.05 a
Isorhamnetin	55.13 ± 41.83 a	31.46 ± 29.14 a	44.17 ± 56.74 a	86.21 ± 58.58 a	39.55 ± 40.86 a	7.26 ± 8.51 a	43.33 ± 40.38 a	38.45 ± 34.93 a
Apigenin	5.22 ± 0.49 b	1.31 ± 0.02 b	0.92 ± 0.12 b	12.33 ± 4.40 a	2.66 ± 0.65 b	0.89 ± 0.52 b	0.60 ± 0.02 b	0.37 ± 0.11 b
Luteolin-7-*O*-glucoside	25.13 ± 17.69 a,b	15.93 ± 8.77 b	70.33 ± 25.55 a	8.87 ± 0.91 b	23.46 ± 3.34 a,b	8.21 ± 10.76 b	18.81 ± 1.85 b	13.33 ± 7.30 b
Procyanidin B1	137.45 ± 4.11 d	181.24 ± 6.81 c	121.19 ± 5.98 d,e	271.30 ± 5.66 a	230.76 ± 8.57 b	207.70 ± 5.60 b,c	97.64 ± 3.71 e	187.28 ± 7.50 c
Chlorogenic acid	394.82 ± 9.61 d	595.98 ± 14.31 c	438.99 ± 15.16 d	897.22 ± 44.37 b	1222.08 ± 45.34 a	944.42 ± 38.45 b	280.31 ± 11.30 e	676.87 ± 15.66 c
Caffeic acid	90.26 ± 1.92 d	106.81 ± 3.60 c,d	104.43 ± 2.92 c,d	143.17 ± 5.66 b	187.03 ± 6.03 a	139.66 ± 2.81 b	67.76 ± 2.08 e	109.85 ± 3.28 c
*p*-Coumaric acid	12.57 ± 0.28 d	23.08 ± 0.83 a,b	20.48 ± 0.54 b	12.89 ± 0.37 d	25.11 ± 0.22 a	20.59 ± 0.43 b	9.50 ± 0.13 e	16.94 ± 0.42 c
Ferulic acid	6.71 ± 0.03 b,c	5.66 ± 0.03 c	8.39 ± 0.14 b	7.56 ± 0.10 b	10.73 ± 0.16 a	11.61 ± 0.26 a	6.11 ± 0.03 c	7.56 ± 0.08 b
Neochlorogenic acid	43.39 ± 3.07 b	42.77 ± 3.02 b	27.05 ± 1.91 c	27.82 ± 1.97 c	82.74 ± 4.24 a	32.48 ± 2.30 c	23.85 ± 1.69 c,d	14.35 ± 1.02 d
4-*O*-Caffeoylquinic acid	47.13 ± 3.33 b,c	47.91 ± 3.39 b	31.11 ± 2.20 d	35.54 ± 2.51 c,d	84.23 ± 5.96 a	45.24 ± 3.20 b,c	25.77 ± 1.82 d,e	15.91 ± 1.13 e
3,5-Dicaffeoylquinic acid	677.35 ± 42.43 d	1061.69 ± 56.57 c	662.36 ± 42.43 d	1601.87 ± 84.85 b	1975.90 ± 127.28 a	1689.49 ± 99.00 b	462.78 ± 28.28 d,e	233.83 ± 14.14 e
3,4-Dicaffeoylquinic acid	37.14 ± 2.63 c	58.17 ± 4.11 a,b	67.71 ± 2.83 a	62.59 ± 1.41 a,b	62.58 ± 4.43 a,b	51.56 ± 3.65 b	56.24 ± 2.83 a,b	28.32 ± 2.00 c

Different letters represent statistically significant differences (*p* < 0.05) between identified phenolic compounds within the same column; ND—not detected.

**Table 6:** UPLC-ESI-MS/MS (negative ionization mode) data of flavonoids and phenolic acids determined in extracts of *L. caerrulea* fruits.

Compounds	Retention Time, min	[M-H]^−^ (*m*/*z*)	Other Ions (*m*/*z*)	Cone Voltage, V	Collision Energy, eV
Rutin	5.06	609	300	70	38
Isoquercitrin	5.28	463	300	52	28
Quercitrin	5.68	447	300	50	26
Quercetin	6.86	301	151	48	20
Isorhamnetin	7.60	315	300	44	22
Apigenin	7.38	269	117	54	36
Luteolin-7-*O*-glucoside	5.31	447	285	66	26
Procyanidin B1	3.44	577	289	50	20
Chlorogenic acid	3.52	353	191	32	14
Caffeic acid	3.89	179	107	36	22
*p*-Coumaric acid	4.73	163	93	28	22
Ferulic acid	5.18	193	134	32	18
Neochlorogenic acid	2.04	353	191	32	14
4-*O*-Caffeoylquinic acid	3.67	353	191	32	14
3,5-Dicaffeoylquinic acid	5.62	515	353	50	25
3,4-Dicaffeoylquinic acid	5.79	515	353	50	25

**Table 7:** Antioxidant activity (µmol/g, TE DW (Trolox equivalents)) and total amounts of proanthocyanidins (mg/g, DW) in cultivars of *L. caerulea* fruits.

Cultivars	Total Proanthocyanidins	ABTS	CuPRAC	FRAP
’Amphora’	2.16 ± 0.03 a	110.10 ± 4.70 a,b,c,d	697.61 ± 30.86 a	237.27 ± 10.91 c
’Iga’	1.06 ± 0.01 d	92.96 ± 46.65 b,c,d	592.51 ± 14.80 a,b	188.32 ± 11.04 c,d
’Indigo Gem’	1.13 ± 0.02 d	192.34 ± 27.95 a	579.92 ± 76.19 a,b,c	707.60 ± 23.81 a
’Leningradskij Velikan’	2.13 ± 0.01 a	170.54 ± 6.66 a,b	565.84 ± 50.69 a,b,c	146.54 ± 8.01 d,e
’Nimfa’	1.46 ± 0.01 b	45.54 ± 22.57 d	670.24 ± 42.02 a,b	208.68 ± 17.26 c,d
’Tola’	0.83 ± 0.004 e	150.06 ± 11.86 a,b,c	190.97 ± 55.37 d	107.66 ± 1.11 e
’Tundra’	1.32 ± 0.03 c	97.04 ± 2.13 b,c,d	515.94 ± 4.77 b,c	348.28 ± 12.50 b
’Wojtek’	0.87 ± 0.06 e	60.82 ± 20.84 c,d	413.11 ± 32.98 c	153.32 ± 27.72 d,e

Different letters represent statistically significant differences (*p* < 0.05) between values within the same column.

## Data Availability

All datasets generated for this study are included in the article.
